# Evaluation of tourism competitiveness and analysis of obstacle factors in border counties of Northeast China

**DOI:** 10.3389/fpubh.2026.1874771

**Published:** 2026-08-31

**Authors:** Yue Gong, Shan Man, Wei Liu

**Affiliations:** College of Landscape Architecture, Northeast Forestry University, Harbin, China

**Keywords:** border tourism, CRITIC–VIKOR model, environmental health, public service support, sustainable tourism development, tourism competitiveness

## Abstract

Border counties often face challenges in transforming tourism resources into sustainable development advantages due to constraints in economic conditions, accessibility, public services, and environmental quality. However, existing tourism competitiveness studies have mainly focused on resource endowment and economic performance, with limited attention to environmental health and public service support as foundations for sustainable tourism development. This study develops a multidimensional framework incorporating Tourism Economic Development, Tourism Resource Attractiveness, Environmental Health, Accessibility and Supporting Infrastructure, and Tourism Service and Public Service Support to evaluate tourism competitiveness in 33 border counties of Northeast China from 2014 to 2023. The Criteria Importance Through Intercriteria Correlation (CRITIC) weighting method and the VIKOR evaluation model were used to measure tourism competitiveness, and an obstacle degree model was applied to identify key constraints and their evolution. The results show that tourism competitiveness in Northeast China’s border counties generally improved, although disparities among counties remained pronounced. Competitiveness levels shifted upward overall, with a gradual increase in high-competitiveness counties distributed across multiple locations in all three provinces. The dominant constraints also shifted from inadequate Tourism Service and Public Service Support toward limitations in Tourism Economic Development, resource transformation, and accessibility. Environmental Health was less frequently identified as a dominant obstacle but remained an important foundation for sustainable tourism development by supporting destination quality and resilience. This study provides insights into sustainable border tourism development by integrating Environmental Health and public service dimensions into tourism competitiveness assessment.

## 1 Introduction

Border regions occupy distinctive geographical and strategic positions and play important roles in promoting regional economic cooperation, ecological conservation, and social development (1, 2). Compared with regional centers, however, border regions commonly face limited transport accessibility, relatively weak industrial foundations, population loss, and insufficient public service provision. These constraints reduce regional development capacity and undermine long-term sustainability. Identifying new development drivers, strengthening overall regional competitiveness, and improving resident well-being have therefore become important issues in border-region research.

In recent years, tourism has increasingly been regarded as an important pathway for economic restructuring and sustainable development in border regions because of its extensive industrial linkages, employment effects, and potential compatibility with ecological conservation (3). Tourism development can promote industrial upgrading and regional economic growth while supporting improvements in infrastructure, public services, and environmental protection. In border regions with abundant resources but relatively weak economic foundations, tourism has consequently become an important sector for economic growth, improved quality of life, and regional revitalization.

Tourism competitiveness provides an integrated measure of a destination’s resource base, development capacity, and supporting conditions. As tourism shifts toward higher-quality development, competitiveness assessments have expanded beyond resource advantages and economic performance to encompass broader enabling conditions. The determinants of tourism competitiveness nevertheless vary across regions. In border areas, economic conditions, resource endowment, accessibility, environmental quality, and public service provision jointly shape the capacity for sustained tourism development. An evaluation framework tailored to the characteristics of border regions is therefore needed.

Despite extensive research on tourism competitiveness, several gaps remain. First, existing evaluation systems generally emphasize conventional factors such as tourism resources, economic development, and infrastructure, while giving less attention to Environmental Health and public services as foundations for sustainable tourism. In the post-pandemic context, environmental quality, health safeguards, and public service capacity affect not only visitor experience but also destination risk-response capacity and long-term resilience. Second, most studies focus on countries, provinces, or major tourism cities, whereas border counties, which combine resource advantages, ecological sensitivity, and development constraints, have received less systematic attention. Third, conventional statistical data provide limited coverage of county-level tourism activity, and the use of multi-source data in integrated county-level assessments remains underdeveloped.

Against this background, this study examines 33 border counties in the three northeastern provinces of China. By integrating official statistics, business registration records, internet data, and policy documents, it constructs a tourism competitiveness framework comprising five dimensions: Tourism Economic Development, Tourism Resource Attractiveness, Environmental Health, Accessibility and Supporting Infrastructure, and Tourism Service and Public Service Support. The Criteria Importance Through Intercriteria Correlation (CRITIC) weighting method and the VIKOR evaluation model are used to assess changes in tourism competitiveness from 2014 to 2023, and the obstacle degree model is subsequently applied to identify the principal constraints on competitiveness improvement. The study seeks to extend research on border tourism competitiveness and provide evidence for high-quality tourism development and sustainable governance in border regions.

## 2 Literature review

Tourism competitiveness refers to a destination’s capacity to attract visitors continuously, create tourism value, and sustain long-term competitive advantages. Its theoretical foundations largely derive from regional competitive advantage theory. Porter’s diamond model argues that regional competitive advantage is shaped not only by factor endowments but also by demand conditions, related and supporting industries, firm strategy, and government action (4). This perspective suggests that destination competitiveness depends on both tourism resources and the wider development environment. Ritchie and Crouch subsequently introduced competitive advantage theory into tourism studies and proposed that destination competitiveness is influenced by natural and cultural resources, supporting resources, infrastructure, destination management, policy and planning, and qualifying determinants (5). Dwyer and Kim further developed an integrated model that combined endowed resources, supporting resources, demand conditions, destination management, and situational conditions, thereby shifting tourism competitiveness research from resource-based assessment toward integrated capability assessment (6). Tourism competitiveness has thus come to be understood as the combined outcome of resource conditions, economic foundations, service systems, and governance capacity rather than a simple measure of resource abundance or tourism industry size (7, 8).

The development of sustainable tourism and tourism resilience research has further broadened the meaning of tourism competitiveness. An increasing number of studies regard competitiveness as reflecting not only a destination’s capacity to attract visitors and generate economic returns but also its ability to support tourism over time (9, 10). Beyond resource endowment and economic conditions, accessibility, ecological quality, public services, health protection, and regional governance have become important dimensions of contemporary tourism competitiveness (11, 12). In the post-pandemic era, heightened visitor concern about environmental quality, public health protection, and safety resilience has encouraged a shift from a conventional resource–economy perspective toward a more integrated resource–economy–environment–service framework (13, 14). Incorporating Environmental Health and public service support therefore extends, rather than alters, the established concept of tourism competitiveness by accounting for the enabling conditions of sustainable destination development (15–17).

Tourism competitiveness is produced through the interaction of multiple factors (18). Resource abundance alone does not guarantee strong competitiveness; the conversion of resources into competitive advantages also depends on economic foundations, accessibility, service capacity, environmental quality, and governance support (11, 12, 16–19). Economic development supports tourism infrastructure, product development, marketing, and investment and therefore reflects a destination’s economic foundation and market vitality (15, 16). Tourism Resource Attractiveness encompasses natural and cultural resources, A-rated Tourist Attractions, Place-specific Tourism Attractions, online attention, and the market appeal created through destination image communication (9, 10, 20, 21). Accessibility and Supporting Infrastructure connect tourism supply with visitor markets. For border counties in particular, entry/exit points and airport accessibility directly influence visitor mobility, cross-regional cooperation, and market expansion (19, 22).

Environmental Health and public service support warrant greater attention in contemporary assessments of tourism competitiveness. Favorable ecological conditions and air quality can improve destination comfort and attractiveness, while environmental sanitation affects visitors’ perceptions of safety and travel experience (23–26). In the context of sustainable tourism and post-pandemic recovery, increased concern about health, safety, and environmental quality has made Environmental Health an important foundation of long-term destination competitiveness (13, 24, 25). Its influence, however, varies across regions and stages of development and may not constitute the dominant constraint in every destination (25, 26). Tourism services such as accommodation, catering, and travel agencies directly affect visitor experience and the conversion of tourism activity into consumption, whereas medical services, government attention, and public service capacity contribute to destination safety, service quality, and governance resilience (27). In border counties with relatively limited public services, both tourism services and public service support affect visitor experience and the stability and sustainability of tourism development (27).

Because tourism competitiveness encompasses economic, resource, environmental, accessibility, and service dimensions, its evaluation is inherently a multi-criteria decision-making problem. Previous studies have applied the analytic hierarchy process, entropy weighting, the Technique for Order Preference by Similarity to Ideal Solution (TOPSIS), VIKOR, and gray relational analysis (28–30). Objective weighting and multi-criteria decision models have been widely used because they can reduce reliance on subjective judgment and improve comparability. Unlike entropy weighting, which primarily determines weights from indicator dispersion, the Criteria Importance Through Intercriteria Correlation (CRITIC) method considers both indicator variability and inter-indicator correlation, allowing weights to reflect information intensity and conflict (31). VIKOR, derived from the Serbian term VIseKriterijumska Optimizacija I Kompromisno Resenje, produces a compromise ranking by jointly considering group utility and individual regret and is suited to evaluation problems involving trade-offs among multiple objectives (32). Combining CRITIC with VIKOR therefore provides an objective weighting basis and a compromise evaluation of county-level tourism competitiveness. The obstacle degree model further integrates indicator weights with deviations from the optimal state to identify constraints on competitiveness and explain differences among counties (33–35).

Overall, existing research provides substantial theoretical and methodological foundations for tourism competitiveness assessment, but several areas require further development. Environmental Health and public service support remain underrepresented in many evaluation systems, limiting their ability to capture the enabling conditions of sustainable destination development. Border counties also remain insufficiently studied despite their distinctive combination of resource advantages, ecological sensitivity, and development constraints. In addition, limited county-level tourism statistics constrain the use of multi-source data in dynamic assessment, while the evolution of competitiveness obstacles has not been fully examined. A multidimensional framework is therefore needed to identify both changes in border tourism competitiveness and the mechanisms that constrain its improvement.

## 3 Study area, materials and methods

### 3.1 Study area

Northeast China’s border areas encompass diverse tourism resources associated with border trade, ethnic cultures, and ecological tourism, giving them distinct resource endowment advantages. However, most border counties face challenges common to border tourism destinations worldwide, including low efficiency in tourism resource development, limited market vitality, inadequate infrastructure, insufficient cross-border cooperation, and tensions between ecological conservation and resource development. Taking the 33 county-level administrative units in Northeast China’s border areas as a case study enables the identification of the spatiotemporal differentiation of border tourism competitiveness and the key obstacle factors constraining its quality improvement and upgrading. This case study can provide evidence to inform tourism development strategies for border regions in China and elsewhere. Based on the Administrative Division Handbook of the People’s Republic of China for 2014–2023 and the criteria used to identify border counties, all 33 border county-level administrative units in Liaoning, Jilin, and Heilongjiang provinces were included in the study. For consistency, counties, autonomous counties, county-level cities, and municipal districts are collectively referred to hereafter as border counties. The 18 units in Heilongjiang Province are Luobei County, Suibin County, Raohe County, Mishan City, Hulin City, Jidong County, Jiayin County, Muling City, Suifenhe City, Dongning City, Tongjiang City, Fuyuan City, Aihui District, Xunke County, Sunwu County, Huma County, Tahe County, and Mohe City. The 10 units in Jilin Province are Ji’an City, Hunjiang District, Linjiang City, Fusong County, Changbai Korean Autonomous County, Tumen City, Longjing City, Hunchun City, Helong City, and Antu County. The five units in Liaoning Province are Zhenxing District, Yuanbao District, Zhen’an District, Donggang City, and Kuandian Manchu Autonomous County.

**Figure 1:**
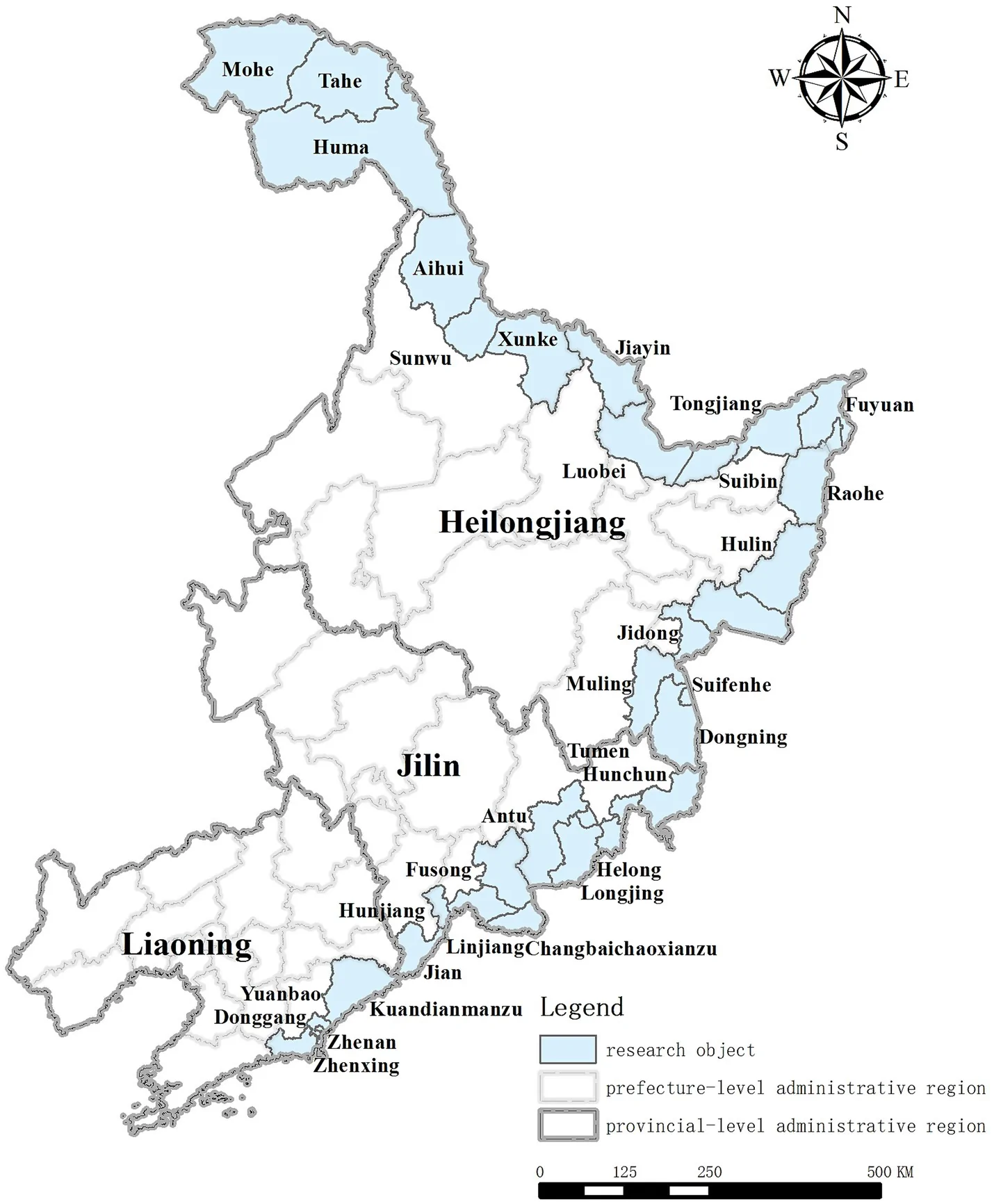
Study area map.

The study area extends from 121°11′ to 135°05′E and from 38°43′ to 53°33′N and contains approximately 4,600 km of international border. It forms an important spatial unit for China’s overland connections with Russia and the Democratic People’s Republic of Korea and occupies a strategic position in Northeast Asia. The area’s diverse physical geography includes mountains, forests, rivers, and coastal environments. Representative natural attractions include volcanic landforms in the Changbai Mountain area, high-latitude ice and snow landscapes around Mohe, forest and wetland ecosystems along the Lesser Khingan Mountains, and coastal and river-confluence landscapes in the Dandong area. Its human geography is characterized by interactions among border cultures and ethnic traditions, including China–Russia border culture, Korean ethnic culture, revolutionary heritage, and port-corridor culture, which together form a continuous cultural landscape along the border (Figure 1).

### 3.2 Evaluation framework and data sources

Drawing on previous research and the characteristics of tourism development in border counties, this study constructs a tourism competitiveness framework comprising five dimensions: Tourism Economic Development, Tourism Resource Attractiveness, Environmental Health, Accessibility and Supporting Infrastructure, and Tourism Service and Public Service Support. Nineteen indicators are used to measure tourism competitiveness in the study area (refer to Table 1).

**Table 1:** Composition of the tourism competitiveness evaluation framework.

Primary indicator	Secondary indicator	Code	Data source
Tourism economic development	GDP per capita	A_1_	County Statistical Yearbook
	GDP growth rate	A_2_	County Statistical Yearbook
	Share of tertiary industry	A_3_	County Statistical Yearbook
	tourism enterprises	A_4_	Enterprise registration information
Tourism resource attractiveness	A-rated Tourist Attractions	B_1_	Provincial Department of Culture and Tourism
	Place-specific Tourism Attractions	B_2_	Online data
	Social-media-popular Attractions	B_3_	Online data
	Online attention	B_4_	Baidu Search Index
Environmental health	Annual temperature range	C_1_	China Meteorological Administration
	Green coverage	C_2_	County Statistical Yearbook
	PM2.5	C_3_	National Tibetan Plateau Scientific Data Center
	Harmless waste treatment	C_4_	County Statistical Yearbook
Accessibility and supporting infrastructure	Number of entry/exit points	D_1_	Amap
	Distance to the nearest airport	D_2_	Amap
Tourism service and public service support	Hospital beds	E_1_	County Statistical Yearbook
	Hotels	E_2_	Enterprise registration information
	Restaurants	E_3_	Enterprise registration information
Tourism Service and Public Service Support includes five indicators, namely E1 through E5	Travel agencies	E_4_	Enterprise registration information
	Government attention	E_5_	Government websites

#### 3.2.1 Construction of the evaluation framework

Tourism economic development: the tourism economy emerges once regional socioeconomic development reaches a certain level. Its evolution generally depends on the coordinated support of regional macroeconomic conditions, the existing industrial base, and related supporting industries (5, 6, 28, 29). This study selected four indicators to embody tourism economic development: GDP per capita, GDP growth rate, share of the tertiary industry, and number of tourism enterprises. GDP per capita and GDP growth rate represent the level and trajectory of economic development, respectively; the share of the tertiary industry reflects the service orientation of the industrial structure; and the number of tourism enterprises indicates the scale of tourism supply and the vitality of market entities. Because county-level data on tourism revenue and visitor arrivals are severely incomplete, the number of tourism enterprises is used as a proxy for the scale of the tourism industry.

Tourism resource attractiveness: tourism resource attractiveness depends on resource endowments and the foundation for resource development, as well as destination image communication and market attention (5, 6, 18, 28). In the digital era, social media platforms and tourists’ online interactions have reshaped perceptions of destination image, thereby influencing travel decisions, on-site behavior, and tourism-related economic activity at destinations (20, 21). In light of this, we selected four indicators to represent tourism resource attractiveness: A-rated Tourist Attractions, Place-specific Tourism Attractions, Social-media-popular Attractions, and Online Attention. A-rated Tourist Attractions represent the established tourism resource base; Place-specific Tourism Attractions identify resources with border, ice, snow, and other local characteristics; and Social-media-popular Attractions and Online Attention reflect emerging attractions and destination visibility in the context of social media communication.

Environmental health: environmental quality, ecological sustainability, and health-supporting conditions have a substantial influence on tourist experience and well-being (8, 14, 17, 24, 25). This study selected four indicators to characterize environment health: annual temperature range, green coverage, annual mean PM2.5 concentration, and harmless waste treatment. Annual temperature range represents climatic stability, green coverage reflects urban ecological development, PM2.5 concentration indicates air quality, and harmless waste treatment reflects environmental sanitation governance.

Accessibility and supporting infrastructure: transportation connects tourism destinations with tourist source markets and facilitates the movement and allocation of people, goods, and information within regional tourism economies. Its level of development directly affects the efficiency and effectiveness of tourism economic activities (6, 19, 22). This study selected two indicators to denote accessibility: the number of entry/exit points and distance to the nearest airport. The former indicates the strength of a county’s external transport connections, while the latter reflects access to the regional air transport network.

Tourism service and public service support: tourism destination service capacity strongly influences tourist experience and satisfaction, while service quality and the scale of provision provide essential support for sustainable regional tourism development (6, 16, 17, 27). This study selected five indicators to manifest tourism service provision and public service support: hospital beds, hotels, restaurants, travel agencies, and Local Government Attention. Hospital beds represent medical assistance and public service capacity; hotels, restaurants, and travel agencies indicate the supply of tourism reception services; and government attention reflects the priority and policy support accorded to tourism development.

#### 3.2.2 Data sources

The study covers 2014–2023, and data collection was completed between January and March 2026. Four categories of data were used.

(1) Statistical yearbook data were obtained primarily from the China County Statistical Yearbook and the statistical yearbooks of Liaoning, Jilin, and Heilongjiang provinces. Data were assigned to the year to which the statistics referred rather than the publication year of the yearbook. GDP per capita, GDP growth rate, share of the tertiary industry, green coverage, harmless waste treatment, hospital beds, and related indicators were compiled for their corresponding statistical years.
(2) Culture and tourism department data were obtained from lists of A-rated Tourist Attractions published by the competent provincial authorities. The year in which each attraction was first awarded an A rating was traced, and 1A–5A attractions were assigned scores of 1–5, respectively. The scores of all A-rated Tourist Attractions in each county were summed at the end of each year to obtain the annual rating-weighted score.
(3) Geographic information and environmental monitoring data were obtained from the China Meteorological Administration, relevant environmental authorities, and geographic information platforms. Accessibility data were collected through Amap. Entry/exit points included conventional railway and high-speed railway stations; publicly available information on station openings and closures was used to reconstruct the number of operating stations at each year-end. Distance to the nearest airport was measured as the straight-line distance from the county government seat to the nearest airport operating in the corresponding year. Annual temperature range was calculated from weather-station observations as the difference between the highest and lowest monthly mean temperatures within a year. PM2.5 data were obtained from the National Tibetan Plateau Scientific Data Center, and annual county-level means were extracted in ArcGIS using county boundaries.
(4) Supplementary multi-source data included business registration records, online platform data, and planning documents. Data on tourism enterprises, travel agencies, hotels, and restaurants were obtained from Tianyancha in February 2026. Industries were screened according to the Statistical Classification of Tourism and Related Industries (2018) and the Industrial Classification for National Economic Activities (GB/T 4754–2017), and registration dates and operating status were used to reconstruct the number of active firms at each year-end. Data on Place-specific Tourism Attractions and Social-media-popular Attractions were collected programmatically from public Xiaohongshu search results in January 2026 using the query “county name + tourism.” Post dates and the construction or opening dates of attractions were used to determine the first year in which each attraction entered the annual dataset. Non-A-rated attractions with border, ice, or snow characteristics were classified as Place-specific Tourism Attractions, and the remainder were classified as Social-media-popular Attractions. Online Attention was measured using the Baidu Search Index. Searches covered January 1 to December 31 of each year and used county names and major attraction names as keywords. The overall search index was used, and the highest value among the keywords was selected as the county’s annual representative value and aggregated over the year. Government attention was measured as the frequency of the term “tourism” in municipal-level 12th, 13th, and 14th Five-Year Plans for National Economic and Social Development, corresponding to 2014–2015, 2016–2020, and 2021–2023, respectively.

To improve historical comparability across data sources, county names, administrative affiliations, indicator definitions, and annual cutoff rules were standardized. Statistical indicators were assigned to their actual reference years; enterprise, attraction, landscape, and transport facility counts were measured as year-end stocks; the Baidu Search Index covered complete calendar years; meteorological and environmental indicators were measured annually; and planning-document indicators remained constant within each Five-Year Plan period. Missing values were limited mainly to hospital beds and harmless waste treatment in some counties before 2019 and GDP per capita in a small number of counties before 2016. Internal gaps bounded by valid observations were filled through linear interpolation, while missing values at the endpoints of the study period were estimated with reference to changes in adjacent years. Interpolated values accounted for less than 10% of all data cells.

### 3.3 Methods

To reduce the influence of extreme values and right-skewed distributions, indicators A_1_, A_4_, B_1_, B_3_, B_4_, E_1_, E_2_, E3, and E_4_ were transformed using ln(x + 1) before modeling; the remaining indicators retained their original forms. C_1_, C_3_, and D_2_ were treated as negative indicators, whereas the other 16 indicators were treated as positive indicators. All indicators were subsequently oriented in the same direction and normalized using min–max normalization.

#### 3.3.1 Criteria importance through intercriteria correlation (CRITIC) weighting method

The CRITIC method was used to determine objective indicator weights and reduce the influence of subjective weighting (31). CRITIC considers both the variability of each indicator and its conflict with other indicators, thereby reflecting the amount of information it contains. A larger standard deviation indicates greater discriminatory power, while weaker correlations with other indicators indicate more independent information and therefore a higher weight.

After the raw indicators were standardized, the standard deviation s_j_ and inter-indicator conflict of indicator j were calculated as follows Equation 1:

$$C_{j}=s_{j}\sum_{k=1}^{m}(1-r_{\mathrm{jk}})$$ (1)

where r_jk_ is the correlation coefficient between indicators j and k, m is the total number of indicators, and C_j_ is the information content of indicator j. A larger C_j_ indicates that the indicator provides greater discrimination among evaluation units.

The weight of each indicator was then calculated as follows Equation 2:

$$w_{j}=\frac{C_{j}}{\sum_{j=1}^{m}C_{j}}$$ (2)

where w_j_ is the weight of indicator j.

#### 3.3.2 VIKOR evaluation model

After the indicator weights were determined, the VIKOR model was used to evaluate tourism competitiveness in the border counties of Northeast China (32). VIKOR is a multi-criteria compromise-ranking method that seeks a solution balancing maximum group utility and minimum individual regret. It is therefore appropriate for integrated evaluations in which multiple indicators differ or conflict.

Let z_ij_ denote the standardized value of indicator j for evaluation unit i, and let f_j_⁺ and f_j_^−^ denote the positive and negative ideal values of indicator j, respectively. Group utility Sᵢ and individual regret Rᵢ were calculated as follows Equations 3, 4:

$$S_{i}=\sum_{j=1}^{m}w_{j}\frac{f_{j}^{+}-z_{\mathrm{ij}}}{f_{j}^{+}-f_{j}^{-}}$$ (3)

$$R_{i}=\max_{j}[w_{j}\frac{f_{j}^{+}-z_{\mathrm{ij}}}{f_{j}^{+}-f_{j}^{-}}]$$ (4)

Sᵢ measures the overall distance between a county and the ideal state across all indicators, whereas Rᵢ represents the largest shortfall on any single indicator.

The VIKOR compromise value Qᵢ was then calculated as follows Equation 5:

$$Q_{i}=v\frac{S_{i}-S^{-}}{S^{+}-S^{-}}+(1-v)\frac{R_{i}-R^{-}}{R^{+}-R^{-}}$$ (5)

where S⁺ and S^−^ are the maximum and minimum values of Sᵢ, respectively; R⁺ and R^−^ are the maximum and minimum values of Rᵢ, respectively; and v is the compromise coefficient. This study sets v = 0.5 to balance group utility and individual regret. A smaller Qᵢ indicates that an evaluation unit is closer to the compromise ideal solution.

For ease of interpretation, Qᵢ was reverse-normalized to obtain a VIKOR-based tourism competitiveness score, with higher values indicating stronger competitiveness Equation 6:

$$C_{i}=1-\frac{Q_{i}-Q_{\min}}{Q_{\max}-Q_{\min}}$$ (6)

where Cᵢ is the VIKOR-based tourism competitiveness score for county-year observation i, and Qmin and Qmax are the minimum and maximum Qᵢ values across all 330 county-year observations, respectively. Cᵢ ranges from 0 to 1. The use of full-sample extrema ensures comparability across years; the resulting score indicates relative competitiveness within the study sample rather than an absolute level of development.

#### 3.3.3 Spatial autocorrelation analysis

To examine whether county-level tourism competitiveness exhibited spatial dependence, Global Moran’s I was used to measure its overall spatial association. A row-standardized first-order Queen contiguity spatial weights matrix was constructed based on the administrative boundaries of the 33 border counties. The spatial weight was set to 1 when two counties shared either a common boundary or a vertex and to 0 otherwise. Global Moran’s I was calculated as follows Equation 7:

$$I=\frac{n}{S_{0}}\frac{\sum_{i=1}^{n}\sum_{j=1}^{n}w_{\mathrm{ij}}(x_{i}-\bar{x})(x_{j}-\bar{x})}{\sum_{i=1}^{n}{\left(x_{i}-\bar{x}\right)}^{2}}$$ (7)

where n is the number of counties; x_i_ and x_j_ are the VIKOR competitiveness scores of counties i and j, respectively; $\bar{x}$ is the mean competitiveness score; w_ij_ is the spatial weight; and $S_{0}=\sum_{i}\sum_{j}w_{\mathrm{ij}}$ . An I value greater than 0 indicates positive spatial association, whereas an I value less than 0 indicates negative spatial association. Statistical significance was assessed using 999 random permutations.

#### 3.3.4 Obstacle degree model

The obstacle degree model was used to identify the factors constraining improvements in county-level tourism competitiveness (33–35). The model combines indicator weights with deviations from the optimal state to quantify the extent to which each factor constrains overall development. For each indicator, the deviation degree was first calculated as the gap between its standardized value and the optimal value. The weighted deviation was then divided by the sum of weighted deviations across all indicators to obtain the obstacle degree Equation 8:

$$O_{\mathrm{ij}}=\frac{w_{j}\times I_{\mathrm{ij}}}{\sum_{j=1}^{m}w_{j}\times I_{\mathrm{ij}}}\times 100\%$$ (8)

where w_j_ is the weight of indicator j, I_ij_ is its deviation degree for evaluation unit i, and m is the total number of indicators. After all indicators have been oriented positively, I_ij_ = 1 − z_ij_. A larger O_ij_ indicates a stronger constraint on tourism competitiveness.

## 4 Results

### 4.1 Tourism competitiveness

#### 4.1.1 Overall characteristics of county-level tourism competitiveness

To characterize the temporal evolution and spatial differentiation of tourism competitiveness across the 33 border counties from 2014 to 2023, CRITIC was used to determine indicator weights and VIKOR was used to calculate tourism competitiveness. Four evenly spaced years—2014, 2017, 2020, and 2023—were selected as representative years. Table 2 reports the five highest- and five lowest-ranked counties in each representative year.

**Table 2:** Tourism competitiveness scores of the five highest- and five lowest-ranked border counties in 2014, 2017, 2020, and 2023.

2014			2017			2020			2023		
County	Score	Rank	County	Score	Rank	County	Score	Rank	County	Score	Rank
Mohe City	0.717	1	Fusong County	0.873	1	Fusong County	0.918	1	Kuandian Manchu Autonomous County	0.998	1
Fusong County	0.711	2	Donggang City	0.830	2	Donggang City	0.860	2	Donggang City	0.971	2
Donggang City	0.705	3	Antu County	0.805	3	Antu County	0.833	3	Fusong County	0.940	3
Antu County	0.701	4	Hunchun City	0.800	4	Hunchun City	0.832	4	Antu County	0.936	4
Suifenhe City	0.687	5	Linjiang City	0.774	5	Kuandian Manchu Autonomous County	0.822	5	Hunchun City	0.918	5
Tongjiang City	0.130	−5	Changbai Korean Autonomous County	0.200	−5	Changbai Korean Autonomous County	0.264	−5	Tongjiang City	0.303	-5
Changbai Korean Autonomous County	0.128	−4	Tongjiang City	0.189	−4	Tongjiang City	0.242	−4	Changbai Korean Autonomous County	0.278	−4
Sunwu County	0.084	−3	Sunwu County	0.149	−3	Sunwu County	0.192	−3	Sunwu County	0.228	−3
Huma County	0.024	−2	Huma County	0.090	−2	Tahe County	0.164	−2	Tahe County	0.225	−2
Tahe County	0.000	−1	Tahe County	0.048	−1	Huma County	0.144	−1	Huma County	0.196	−1

Tourism competitiveness in the border counties of Northeast China generally increased. Based on full-sample weighting and standardization, the mean VIKOR-based tourism competitiveness score rose from 0.459 in 2014 to 0.669 in 2023, an increase of approximately 45.6%. The mean score increased steadily from 2014 to 2022, reaching 0.673 in 2022, before declining slightly to 0.669 in 2023. It nevertheless remained substantially above the initial level, indicating an overall strengthening of the foundations and supporting conditions for border tourism. At the same time, disparities widened: the range of county scores increased from 0.717 in 2014 to 0.802 in 2023, while the maximum score rose from 0.717 to 0.998. Although some lower-competitiveness counties improved, their progress was comparatively slow. The region therefore exhibited simultaneous overall improvement and increasing stratification, with larger gains concentrated in counties characterized by stronger resource endowments, favorable locations, and relatively well-developed service systems.

**Figure 2:**
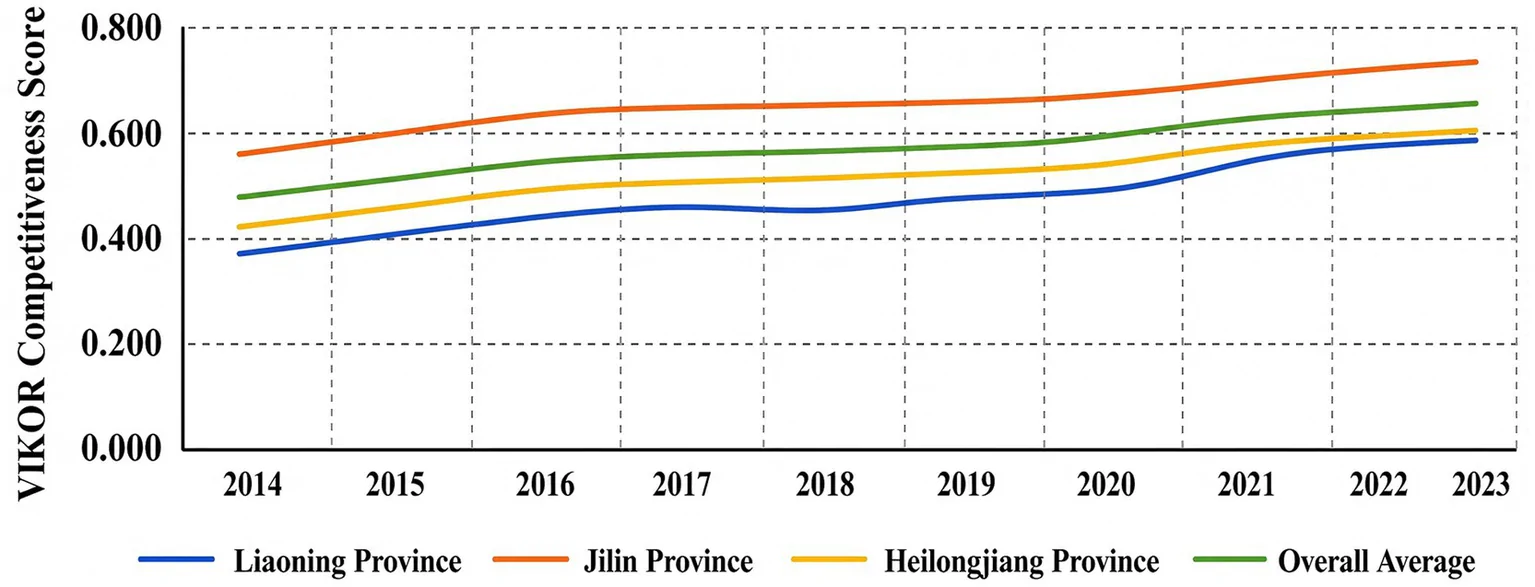
Trends in provincial and overall mean tourism competitiveness scores, 2014–2023.

At the provincial level, border counties in Jilin recorded the highest mean tourism competitiveness score over 2014–2023 (0.686), followed by Heilongjiang (0.541) and Liaoning (0.499) (refer to Figure 2). Jilin maintained a relatively strong position because of the Changbai Mountain and Yanbian tourism resource base and comparatively developed service provision. Heilongjiang displayed substantial internal variation: Mohe, Suifenhe, Mishan, and Dongning performed strongly, whereas several other counties remained constrained. Liaoning showed a spatial pattern in which counties along the Dandong border performed better than districts closer to the urban core.

#### 4.1.2 Spatiotemporal evolution of county-level tourism competitiveness

To examine dynamic differentiation, the 330 county-year tourism competitiveness observations for 2014–2023 were pooled, and the Jenks natural breaks method was used to determine a common four-level classification of high, relatively high, relatively low, and low competitiveness. Applying the same breakpoints to every year ensured interannual comparability.

**Figure 3:**
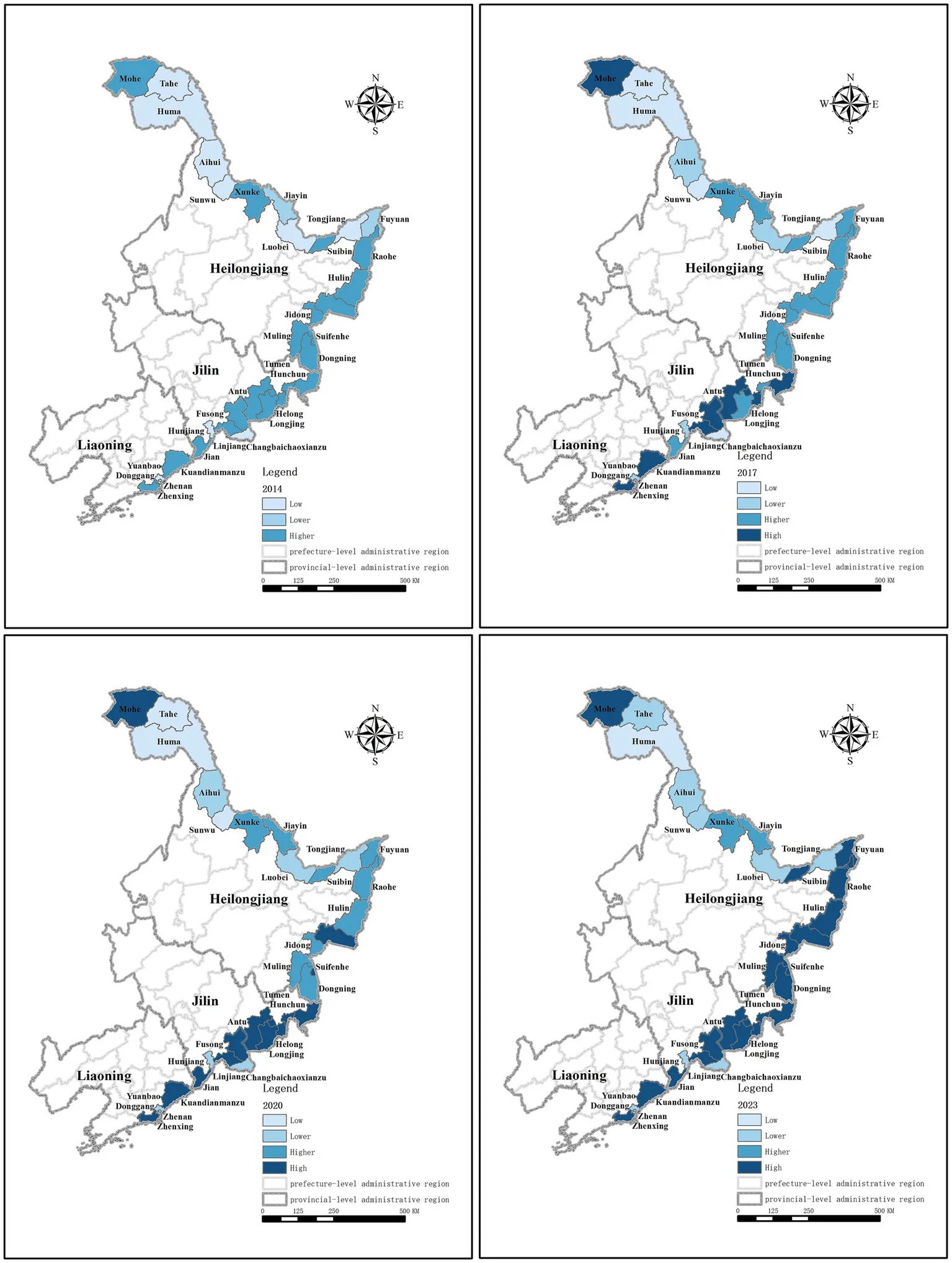
Spatial distribution of tourism competitiveness levels among border counties in 2014, 2017, 2020, and 2023.

The distribution of competitiveness levels shifted upward over the study period (refer to Figure 3). In 2014, no county was classified as high competitiveness; 20 were relatively high, two were relatively low, and 11 were low. In 2017, the corresponding numbers were six, 16, five, and six. In 2020, they were 12, 10, eight, and three. By 2023, 19 counties were classified as high, three as relatively high, eight as relatively low, and three as low. Thus, the number of high-competitiveness counties increased from zero to 19, while the number of low-competitiveness counties declined from 11 to three. The decrease in the relatively high category largely reflected upward movement into the high category rather than an overall deterioration in competitiveness.

Spatially, tourism competitiveness in the border counties of Northeast China was characterized by a gradual increase in the number of high-competitiveness counties and their distribution across multiple locations in all three provinces. In 2014, the overall competitiveness level was relatively low, with no county classified as having high competitiveness. Counties with relatively high competitiveness were distributed across all three provinces, whereas low-competitiveness counties were relatively more numerous in Heilongjiang. In 2017, the six counties classified as having high competitiveness were Fusong, Donggang, Antu, Hunchun, Linjiang, and Longjing, which were mainly located in Jilin’s border areas and the Dandong border area. By 2020, the geographical extent of high competitiveness had expanded further, with Kuandian Manchu Autonomous County, Mohe, and Mishan entering the high-competitiveness group. The spatial distribution began to shift from relative concentration in Jilin toward multiple locations across all three provinces. By 2023, the number of high-competitiveness counties had increased to 19, and 18 of the 20 counties classified as having relatively high competitiveness in 2014 had moved into the high-competitiveness group. Meanwhile, the low-competitiveness group had contracted to Sunwu, Huma, and Tahe, all located in northern Heilongjiang, indicating that the overall improvement had not completely eliminated persistent low-level constraints.

To determine whether the observed classification pattern exhibited statistically significant spatial association, Global Moran’s I was further calculated. The annual Moran’s I values for 2014–2023 were all positive, ranging from 0.095 to 0.176, but none was significant at the 5% level based on the two-sided random permutation tests. These results indicate a weak tendency toward positive spatial association in county-level tourism competitiveness, but no significant or stable global spatial clustering. Overall, the evolution of tourism competitiveness was characterized primarily by an upward shift in the competitiveness structure and an expansion in the geographical distribution of high-competitiveness counties. Nevertheless, some counties remained at relatively low levels, and regional development disparities were not fully eliminated.

### 4.2 Obstacle factors affecting tourism competitiveness

#### 4.2.1 Overall characteristics of obstacle degrees

To identify the principal constraints on tourism competitiveness, the obstacle degree model was applied to each indicator for every county from 2014 to 2023, and the obstacle factors were ranked and compared (refer to Table 3).

**Table 3:** Mean obstacle degrees of individual indicators across border counties in 2014, 2017, 2020, and 2023.

2014		2017		2020		2023	
Indicator	Obstacle degree (%)	Indicator	Obstacle degree (%)	Indicator	Obstacle degree (%)	Indicator	Obstacle degree (%)
E₅	10.31	E₅	10.49	E₅	11.79	B₄	10.35
B₂	8.22	B₂	8.40	B₄	9.39	D₁	10.19
A₃	7.96	D₁	8.31	D₁	9.32	A₃	8.74
D₁	7.87	B₄	8.25	B₂	8.42	B₂	8.29
B₄	7.63	A₃	7.68	A₃	7.98	C₁	7.40
C₃	7.05	A₁	6.54	A₁	7.30	A₁	7.06
A₁	5.54	C₃	5.40	C₂	5.41	E₅	5.62
E₄	4.95	C₂	5.35	E₄	4.70	C₂	5.17
E₂	4.83	E₄	5.05	A₄	4.37	D₂	4.47
A₄	4.78	A₄	4.66	E₂	4.34	E₂	4.37
C₂	4.76	E₂	4.52	D₂	4.06	E₄	4.24
E₃	4.34	E₃	3.88	C₁	3.59	A₄	3.97
C₁	4.30	C₁	3.80	A₂	3.54	A₂	3.84
B₁	3.79	D₂	3.63	E₃	3.53	C₃	3.56
B₃	3.66	A₂	3.47	C₃	3.31	E₃	3.50
D₂	3.13	B₁	3.46	B₃	3.07	B₃	3.23
A₂	2.92	B₃	3.28	B₁	3.02	E₁	3.02
E₁	2.39	E₁	2.50	E₁	2.70	B₁	2.99
C₄	1.56	C₄	1.34	C₄	0.16	C₄	0.00

Based on full-sample mean obstacle degrees, the main constraints were associated with Tourism Service and Public Service Support, Tourism Resource Attractiveness, Accessibility and Supporting Infrastructure, and Tourism Economic Development. Government attention, Online Attention, number of entry/exit points, Place-specific Tourism Attractions, share of the tertiary industry, and GDP per capita ranked highest, with mean obstacle degrees of 9.240, 8.969, 8.922, 8.374, 8.230, and 6.713, respectively. These results identify policy attention and governance, the market visibility of tourism resources, external transport connections, and the local economic base as major factors shaping tourism competitiveness in Northeast China’s border counties.

The leading obstacle factors changed over time. In 2014, government attention had the highest obstacle degree (10.307), while Place-specific Tourism Attractions, share of the tertiary industry, number of entry/exit points, and Online Attention were also prominent. At this early stage, government support, resource development, and industrial capacity were central constraints. By 2023, Online Attention and the number of entry/exit points ranked first and second, with obstacle degrees of 10.353 and 10.187, whereas the obstacle degree of government attention had declined to 5.619. The dominant constraints had therefore shifted from limitations in resource availability and policy attention toward inadequate market recognition of tourism resources and limited external accessibility. Border tourism competitiveness increasingly depended on resource conversion and market connectivity rather than on basic factor inputs alone.

The combined share of the six leading obstacle factors also increased, from approximately 49.03 in 2014 to 52.02 in 2023. This change indicates that the principal constraints became more concentrated among a smaller group of core factors.

#### 4.2.2 Evolution of the obstacle pattern

**Figure 4:**
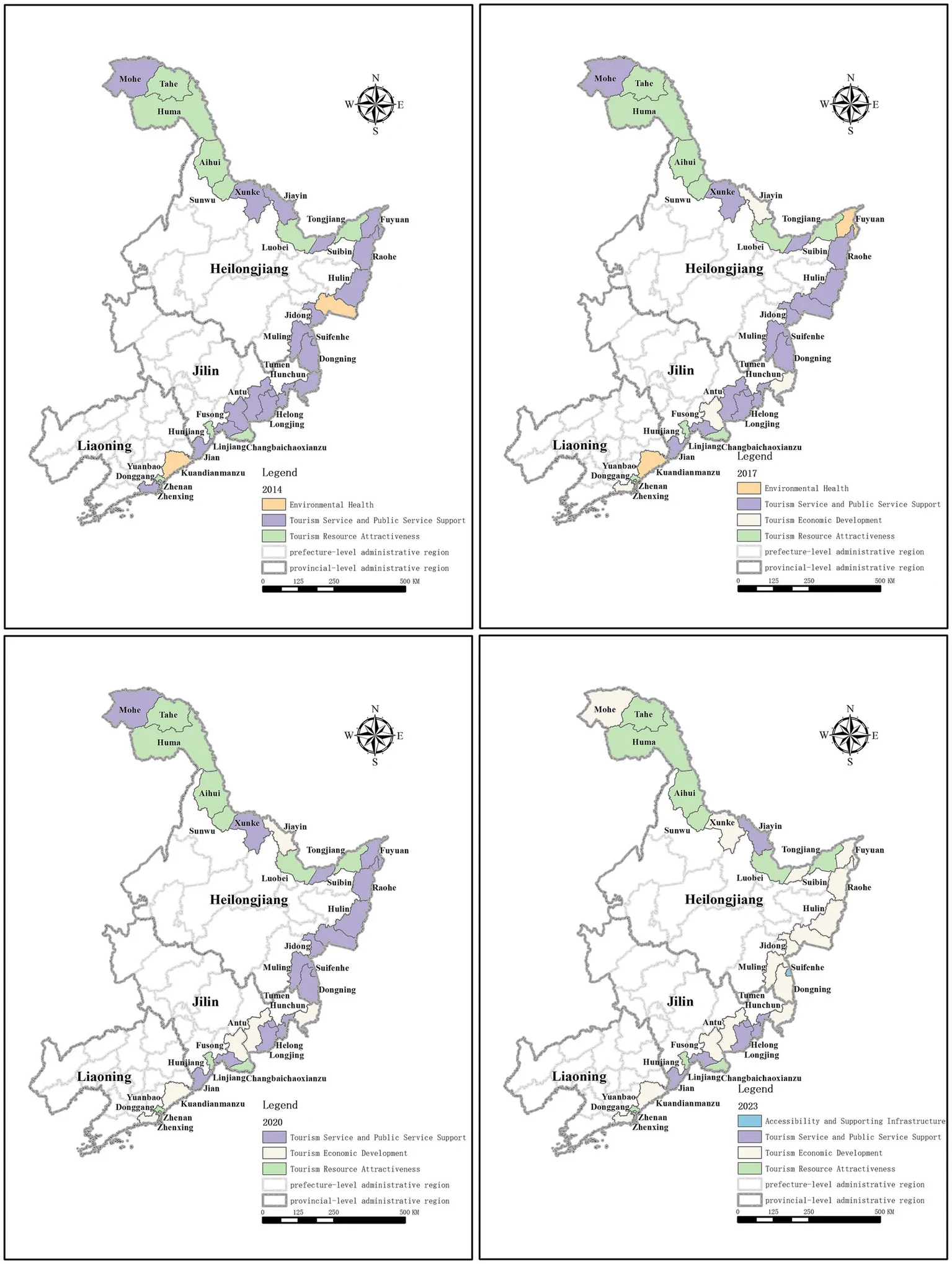
Spatial distribution of dominant obstacle types among border counties in 2014, 2017, 2020, and 2023.

Based on the contributions of the five criterion-level dimensions, the dominant obstacle type was identified for each county and its evolution was examined (refer to Figure 4). The results reveal a stage-dependent transition from early deficiencies in Tourism Service and Public Service Support toward constraints in Tourism Economic Development, indicating a shift from foundation building to industrial conversion.

In 2014, Tourism Service and Public Service Support was the most common dominant obstacle type, followed by Tourism Resource Attractiveness, while only a few counties were primarily constrained by Environmental Health. Border tourism was still at an early capacity-building stage, and weaknesses in reception services, public services, and tourism organization limited competitiveness. Some counties also possessed resource advantages that had not yet been fully developed or marketed, making Tourism Resource Attractiveness an important constraint.

In 2017 and 2020, Tourism Service and Public Service Support remained the leading obstacle type, although the number of counties for which it was dominant declined. At the same time, Tourism Economic Development and Tourism Resource Attractiveness became increasingly prominent. As basic tourism services improved, the principal constraints shifted from inadequate service provision toward weaknesses in industrial foundations and resource conversion.

By 2023, Tourism Economic Development had become the most common dominant obstacle type, followed by Tourism Resource Attractiveness and Tourism Service and Public Service Support, while several counties were constrained primarily by Accessibility and Supporting Infrastructure. Traditional service and infrastructure deficiencies had eased, whereas the economic base, industrial scale, tourism consumption capacity, and external market connections had become more important constraints on further competitiveness gains.

The transition pathways varied across obstacle types. Tourism Resource Attractiveness remained relatively stable and continued to dominate in some counties throughout the study period, indicating that converting resource advantages into tourism competitiveness remains a long-term challenge. Although some counties possess ecological landscapes and border-cultural resources, limited market recognition, product development, and brand influence have prevented these resources from being fully converted into tourism appeal.

Tourism Service and Public Service Support declined markedly as a dominant obstacle, with most transitions occurring toward Tourism Economic Development. Improvements in tourism infrastructure, policy attention, and service capacity alleviated early supply constraints, while weak economic foundations, limited industrial scale, and insufficient endogenous development capacity became more prominent.

Environmental Health was the dominant obstacle in only a small number of counties and was gradually replaced by Tourism Economic Development. Environmental Health therefore operated primarily as a foundational condition rather than a persistent determinant of competitiveness differences. As pollution control, environmental sanitation, and ecological protection improved, their direct constraints weakened, while inadequate economic support became more prominent in some counties.

Overall, the obstacle structure shifted from supply-support constraints toward industrial-development constraints. In the early period, limited tourism services and public service support weakened the foundations of tourism development. As these conditions improved, resource conversion efficiency, economic support, and market connectivity became increasingly influential. Border tourism development thus moved from addressing basic deficiencies toward improving industrial quality and development efficiency.

## 5 Discussion

### 5.1 From resource endowment to resource conversion

The results show that tourism resource endowment does not automatically generate sustained competitive advantages. Resource conversion depends jointly on market recognition, product development, transport connections, and industrial support. During the study period, the dominant obstacles shifted from inadequate service support toward weaknesses in the tourism economy, resource conversion, and market connectivity, suggesting that border tourism moved from improving basic conditions toward enhancing quality and efficiency. Resource-rich but geographically remote counties should therefore improve market conversion through digital communication, destination branding, better accessibility, and industrial integration.

### 5.2 Environmental health and public service support

In addition to conventional tourism competitiveness dimensions, this study incorporates Environmental Health and public service support. Environmental Health was not the leading source of current differences in competitiveness, but it remained a foundational condition for sustainable tourism development. Its role is stage dependent: economic foundations, accessibility, and service capacity often dominate at earlier stages, while environmental quality, ecological comfort, and healthy travel experiences become increasingly relevant as basic conditions improve. Incorporating Environmental Health therefore addresses the limited attention given to environmental quality and public service support in conventional frameworks and provides a broader understanding of long-term destination capacity. Public service support is also particularly important in border counties characterized by small populations and long distances from major markets, because tourism services and public services affect both visitor experience and industry stability. Environmental improvement and stronger public services should therefore form part of long-term competitiveness strategies.

### 5.3 Governance implications for sustainable border tourism

Tourism competitiveness in Northeast China’s border counties cannot be improved through a single development pathway; strategies should respond to local conditions and dominant constraints. High-competitiveness counties should shift from expansion toward quality improvement by developing new tourism products and consumption settings and strengthening digital communication. Counties with intermediate competitiveness should improve resource commercialization and regional connectivity through integrated resource development, coordinated tourism routes, and cross-regional cooperation. Low-competitiveness counties should prioritize accessibility, public service support, and access to market information to reduce visitor entry costs and establish distinctive development pathways. Across all groups, Environmental Health and public service support should be incorporated into long-term tourism strategies, linking ecological improvement, healthy travel experiences, and service-system development with resource conversion and overall efficiency.

### 5.4 Limitations and future research

This study has several limitations. First, data availability at the county level required the use of proxy indicators. Enterprise counts and online attention, for example, cannot fully capture actual business scale or visitor behavior. Future research could integrate mobile signaling data, online travel platform data, and consumption records to improve the measurement of tourism activity. Second, although the CRITIC–VIKOR approach provides an integrated multi-criteria evaluation, the results remain sensitive to indicator selection and model specification. Spatial econometric models, machine-learning methods, and causal identification strategies could be used to examine the mechanisms that generate tourism competitiveness. Third, this study focuses on changes at the county level and gives limited attention to cross-regional tourism connections, visitor-flow networks, and border cooperation. Future work could analyze border tourism competitiveness from the perspective of regional coordination and network relations.

## 6 Conclusion

Using data for 33 border counties in Northeast China from 2014 to 2023, this study applied CRITIC weighting, the VIKOR evaluation model, and the obstacle degree model to examine the spatiotemporal evolution of tourism competitiveness and its constraining mechanisms. The main conclusions are as follows.

First, tourism competitiveness generally increased. The mean county score rose from 0.459 in 2014 to 0.669 in 2023, while internal disparities remained pronounced and the range between the highest and lowest scores widened from 0.717 to 0.802.

Second, Jilin maintained the strongest overall provincial position, Heilongjiang displayed substantial internal variation, and Liaoning’s strongest counties were concentrated along the Dandong border. Fusong, Donggang, Antu, Hunchun, and Kuandian Manchu Autonomous County remained among the stronger counties, whereas Tahe, Huma, and Sunwu remained relatively weak.

Third, the Jenks natural breaks classification showed a marked upward shift in county-level tourism competitiveness during the study period. The number of high-competitiveness counties increased from none in 2014 to 19 in 2023, including two in Liaoning, eight in Jilin, and nine in Heilongjiang. Global Moran’s I values were positive in all years, indicating a weak tendency toward positive spatial association, but none reached statistical significance. This suggests that the increase in high-competitiveness counties primarily reflected expansion across multiple locations in the three provinces rather than significant global spatial clustering.

Fourth, the single-indicator obstacle degrees identified government attention, Online Attention, number of entry/exit points, Place-specific Tourism Attractions, share of the tertiary industry, and GDP per capita as the principal obstacle factors. Over time, the leading constraints became increasingly concentrated in market communication, transport connectivity, and economic support.

Fifth, the dominant obstacle structure shifted from Tourism Service and Public Service Support toward the coexistence of constraints in Tourism Economic Development, Tourism Resource Attractiveness, and Accessibility and Supporting Infrastructure. Tourism Service and Public Service Support declined from its initially dominant position; Environmental Health constraints shifted from pollution control toward environmental quality and healthy destination conditions; and the importance of Tourism Economic Development, Tourism Resource Attractiveness, and Accessibility and Supporting Infrastructure increased. Overall, border tourism development moved from addressing basic factor deficiencies toward a stage emphasizing resource conversion, market connectivity, healthy environments, and integrated efficiency.

## Data Availability

The original contributions presented in the study are included in the article/supplementary material, further inquiries can be directed to the corresponding author.
